# Development of a smart guide wire using an electrostrictive polymer: option for steerable orientation and force feedback

**DOI:** 10.1038/srep18593

**Published:** 2015-12-17

**Authors:** F. Ganet, M. Q. Le, J. F. Capsal, P. Lermusiaux, L. Petit, A. Millon, P. J. Cottinet

**Affiliations:** 1 Université de Lyon - INSA de Lyon – LGEF, 8 rue de la Physique, 69 621 Villeurbanne – France; 2 Pulsalys, 47 Boulevard du 11 Novembre 1918, CS 90170, 69625 Villeurbanne – France; 3 Groupement Hospitalier Edouard Herriot - Chirurgie Vasculaire – Pav. M – France; 4 Université de Lyon – Université Claude Bernard Lyon 1, 8 Avenue Rockefeller Lyon – France

## Abstract

The development of steerable guide wire or catheter designs has been strongly limited by the lack of enabling actuator technologies. This paper presents the properties of an electrostrive actuator technology for steerable actuation. By carefully tailoring material properties and the actuator design, which can be integrated in devices, this technology should realistically make it possible to obtain a steerable guide wire design with considerable latitude. Electromechanical characteristics are described, and their impact on a steerable design is discussed.

Image-guided endovascular interventions have gained in popularity in clinical practice as studies have consistently shown that these minimally invasive interventions are of equivalent or greater efficiency and offer lower mortality rates when compared to traditional open surgical techniques^1^,^2^,^3^. A majority of interventions involve the use of flexible guide wires and catheters that are guided by the surgeon into the appropriate vessels under real-time X-ray imaging^4^,^5^,^6^. Maneuverability of a guide wire for intravascular navigation is the key to reaching the targeted area; the ability to steer a guide wire thus affects to a great extent the duration and success of the procedure. The difficulty of steering and controlling the guide wire increases the risks for complications, including vascular dissection, perforation, and thrombosis^7^. Some of these risks can be offset by systemic heparinization, which is routinely used in clinical endovascular procedures performed today, though this may itself increase the risk of procedural hemorrhage.

For many procedures the guide wire is guided from a safe entrance vessel (e.g., the common femoral artery) to a target that is relatively far away in the body via branched or tortuous vessels^7^. With a traditional guide wire, its tip is steered by manually rotating the wire about its axis and pushing it forward into the desired vessel. After the guide wire has been navigated through several vascular turns, the torque at the proximal end is hindered^7^,^8^. Moreover, it is difficult to manipulate a guide wire tip through sharp turns, for instance when entering a recurrent branch vessel whose origin is directed at an orientation greater than 90 degrees to the parent vessel^9^.

With these evident challenges, various guidance mechanisms for guide wire and catheter navigation have been designed and are in clinical use. Manual and pullwire guidance were the first to be used. Guide wires for manual direction are flexible, small-diameter wires placed through the patent lumens of catheters and are manually navigated into branch vessels before the functional catheter tip to serve as a stable track for the catheter to follow^10^. Manually controlled, shapeable, metallic guide wires of variable stiffness placed through variably stiff plastic catheters have been the mainstay of endovascular catheter guidance under X-ray fluoroscopic imaging for decades, and the number of guide wires and catheters available for specific applications throughout the body is huge^7^,^10^.

Smart material actuators provide an alternative to the classical mechanisms of guidance. Shape memory polymers (SMPs) have during the past 10 years been investigated for this purpose. Direct heat is the most commonly used trigger for an SMP to resume its original shape, although ultraviolet and infrared radiation, electrical current, pH changes, and magnetic fields have also been used^11^. In a study by Buckley *et al.*, inductive heating was used to induce a change in SMP shape by loading ferromagnetic particles into the polymer and exposing it to an alternating magnetic field, as could be used in conjunction with MRI^12^. Shape memory alloys (SMAs) are better studied than SMPs and have a shorter response time, but also a higher density, higher cost, and lower attainable strains^13^.

A shape memory alloy actuated catheter has been designed such that tubes of the SMA were distributed about the central axis of the catheter and could be heated to achieve bending due to shrinking of the SMA material^7^. When the heating stops, the material is naturally cooled and reforms its original shape^7^. Some major considerations when it comes to these methods include safe heating, efficient cooling, the use of many lead wires if the catheter assumes a multi-link style and a complex position control due to the hysteresis^13^. Hydraulic systems have also been investigated; for example, Miles patented a pneumatic or hydraulic catheter design in which tubing runs on either side of a catheter to an elastomeric cylinder^14^. However, the size of such devices makes them complex when it comes to their future utilization with Minimally Invasive Methods (MIS) of surgery.

Ionic Polymer Composite (IPMC) actuators have also been suggested for the design of active catheters or guide wires^15^,^16^,^17^. These actuators can generate large displacements at relatively low electrical field and moderate speed; however, their manufacturing process is often relatively expensive and additional energy is usually consumed in order to hold the actuator in position^18^. Electrostrictive polymer actuators, especially poly(vinylidene fluoride-trifluoroethylene-chlorotrifluoroethylene), namely terpolymer P(VDF-TrFE-CTFE), have shown attractive properties, which make them promising for extensive employment in active guide wire applications^19^. Some of their characteristics include a high energy density, ease of fabrication, and a relatively high strain^20^. However, in order to generate large deformations, electrostrictive polymers require very high electrical fields which block the development of steerable guide wire. Recently, a novel approach enabling to drastically improve the mechanical energy density of an electrostrictive polymer at low electrical fields has been investigated in^21^,^22^. This should make it possible to overcome this technological issue.

Recently, Capsal *et al.* proposed a simple and efficient solution to improve the electromechanical performances of the P(VDF-TrFE-CTFE) fluorinated terpolymer by reducing the required electric field^22^. The results demonstrated that a doping of the polymer with bis(2-ethylhexyl) phthalate (DEHP) plasticizer leads to improved molecular mobility and large Maxwell Wagner Sillars interfacial polarization effects, resulting in decrease Young modulus as well as increase dielectric permittivity of the material^23^. Hence, choosing an adequate polymer matrix/plasticizer combination would enable the generation of large macroscopic dipoles combined with phase heterogeneities while reducing the Young’s modulus of the polymer and increasing its dielectric permittivity.

As a result of the improved dielectric permittivity and the decreased Young modulus, a great enhancement of the low-frequency electrostrictive transverse strain (a twenty-eight-fold increase compared to the neat fluorinated-polymer at 10 V/μm) was achieved. More importantly, compared to traditional composites, the developed plasticizer-polymers exhibited a decreased dielectric breakdown strength which is very important for practical actuator applications^24^,^25^. Consequently, the approach proposed herein, using a low-cost plasticizer bis(2-ethylhexyl) phthalate (DEHP), is promising for the development of new all-organic composites with excellent electrostrictive properties for steerable guide wires.

The present paper is divided into four parts. The first part is dedicated to the requirements and working principal of steerable guide wires. The second presents the material properties and the elaboration of devices used in this work. The third part concerns the medialization of the “smart” guide wire, using finite element modeling. Finally, the last part is dedicated to the analysis of the results.

## Requirements and Working Principals

The requirements for actuators to be used for “smart” guide wires can be very broad. However, the major performances to take into account when designing remotely steerable guide wires are the bending angles achievable by the guide wire, the time to obtain bending, the degree of rotation that can be achieved, and the capacity for miniaturization of the design^26^. The design of the guide wire shaft is a significant factor in determining the formation of curves, the angles of deflection and the levels of steerability. The choice of material determines the level of pushability, torque and flexibility and can be manipulated along the length of the guide wire through a variety of means to achieve the desired results. The objective of this study was to demonstrate the possibility of using an electroactive polymer for a steerable guide wire, and the overriding constraints were related to the following points^26^:

- Typical angle in human vessels.
- Time response.

Hutchins *et al.* realized a study on the typical curvature and angle in human vessels^27^. Measurements were made of parent and branch vessel diameters and of the included angles of branch-points from postmortem human coronary arteries. The results demonstrate no relationship between the branch-angle and the vessel caliber. The included angle between branches varied from 32 degrees to 124 degrees regardless of the relative or absolute vessel calibers. It was hence necessary to develop “smart” wire guides able to generate these bending angle values. Moreover, it is interesting to note that most medical device suppliers propose catheters or guide wires that have been pre-bent between 30° and 120° 28.

**Figure 1:**
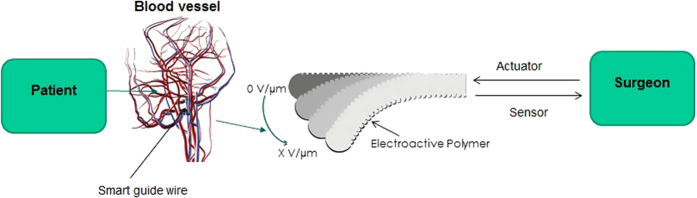
Schematic illustration of the “smart” guide wire application.

The time response of the actuator is directly linked to the physical property of the surgeon, since the “smart” guide wire is controlled by the surgeon as illustrated in Fig. 1. The typical reaction time of humans is about 180 ms^29^. Two effects contribute to this delay. The first is the time that elapses between photons arriving at the retina and the surgeons perceiving that the guide wire is not in a good position (in other words, the time delay in human vision). The second is the time that elapses between nerve impulses leaving the brain and movement in the muscles. There is another delay concerning the electronic control of the guide wire (10 ms), so typically it is necessary that the time response of the actuator does not exceed 100 ms. It is easy to understand this value since visualizing the guide wire tip while navigating vessels is important in order to avoid that the guide wire tip is undesirably forced through a vessel or tissue, causing perforation. Consequently, the time response of the actuator needs to be lower than the time response of the surgeon.

Although the traditional robotic actuators which are currently used in robotic surgical devices, such as electro-magnetic drives and hydraulic/pneumatic machines, have all been extensively investigated and have advanced performances which can, in some aspects, surpass that of humans, they simply do not have the capabilities and diversity required to meet the demand for new actuation systems in the proposed application. They are stiff, noncompliant and mechanically complex, which makes scalability and miniaturization difficult. To meet the technical requirements, electrostrictive polymers could be used for solid-state actuators. They can be realized by sandwiching a polymer dielectric layer between electrodes. When applying an electrical field to the electrodes, the dielectric material contracts due to electrostatic forces and expands in the lateral directions.

**Figure 2:**
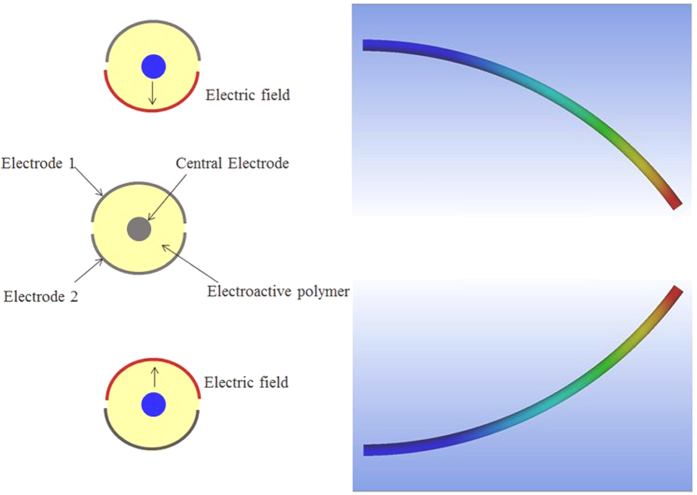
Principle of a “smart” guide wire with 2 degrees of freedom.

This work has focused on the modeling, design and realization of a cylindrical guide wire with two degrees of freedom (DOF). The 2-DOF functionality was achieved by uniquely sectored electrodes on the surface of a cylindrical electrostrictive polymer as illustrated in Fig. 2. Such a design allows the smart guide wire to bend in both directions. In fact, there exists a quadratic relationship between the strain and the electrical field; hence, the generated displacement is independent of the sign of the electrical field. If an electrical field is applied between the central electrode and electrode 1, the polymer is, due to the electrostatic force, compressed in the thickness direction, causing the structure to bend. If the electrical field is applied between the central electrode and electrode 2, the smart catheter bends in the opposite direction.

## Modeling of the Structure

In view of the architecture of the actuator, an analytical approach was deemed too complex for modeling the electromechanical behavior of the “smart” guide wire. Therefore, finite element modeling was chosen. The multi-physics finite element model was developed using Ansys. This tool is used to model the basic physics occurring within an electrostrictive polymer. The models are developed to track the geometric variation of the electroactive polymer in the presence of an electrical field. These variations of geometries are then correlated to a mechanical generated stress^30^.

To produce this model, the geometry of the “smart” guide wire was idealized as a perfect circle with constant electrode shapes. These geometries were created using the built-in Ansys tools. The use of these built-in geometry tools made it possible to input the dimensions as Ansys parameters and easily adjust them throughout the modeling process. To create the three-dimensional shape, a two-dimensional electroactive polymer cross-section was drawn and then extruded to create the length of the guide wire.

The mesh was defined on the face of the EAP and then extended along the length of the EAP. It was designed to be fine near the electrode polymer interface which was accomplished using a distribution boundary condition. This condition guaranteed that the number of elements in this critical region remained constant even when the whole size in the middle was varied. The region was considered critical since it was where the largest changes in concentration were seen.

To perform the simulation, two physics packages were simultaneously used: electro-statics, and solid mechanics^30^. The electro-statics analysis focuses on the input of the electrical field to the EAP, and this physics package is governed by Poisson’s law. The last physics package used in the simulation was the structural mechanics model. The bending in the tube-type EAP was modeled using a linear elastic model. The simulation parameters can be seen in Table 1. Two compositions of the electroactive polymer were used, a neat terpolymer and a modified terpolymer; the data was extracted from the literature^22^ and is presented in Table 2.

**Figure 3:**
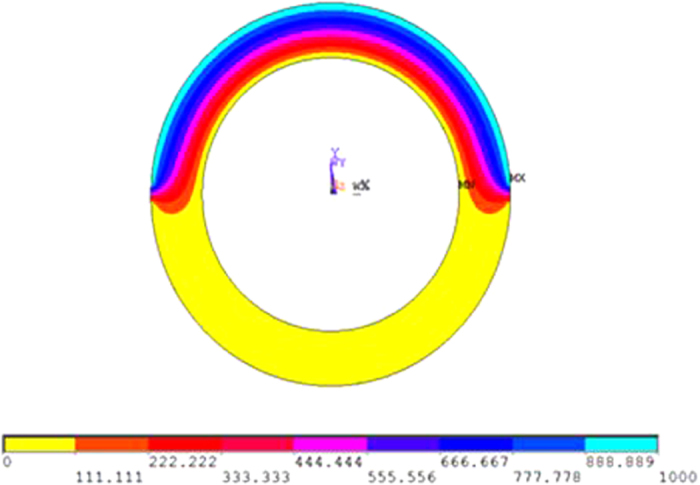
Electrostatic reparation in the in cross-section view.

For the first simulation, an electrical field of 30 V/μm was applied between electrode 1 and the central electrode. The electric potential in the cross-section of the EAP is show in Fig. 3. The electrical field spread across the EAP in a symmetric manner between the central electrode and electrode 1. Due to geometric inconsistencies in the real sample, this is not always the case. The electrical field between electrode 2 and the central electrode appeared to be null, which was logical since no electrical field was applied. This electrode configuration allows a sectoring of the electrical field distribution in the structure. This was necessary for the development of a steerable guide wire.

**Figure 4:**
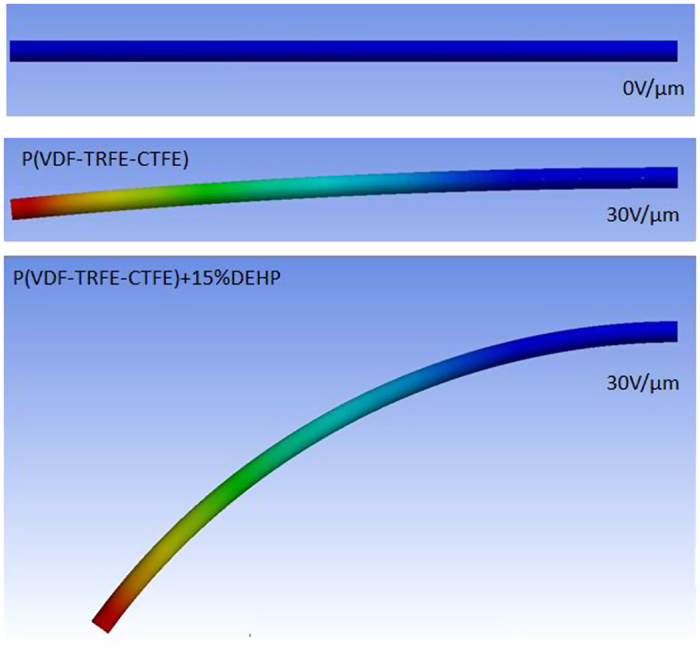
Bending displacement of two compositions of a terpolymer under a constant electrical field of 30 V/μm.

**Figure 5:**
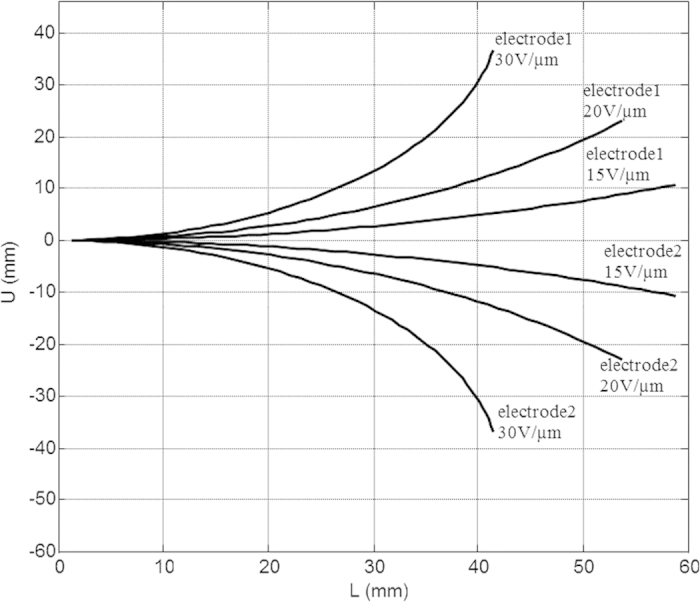
Bending displacement along the guide wire length for a modified terpolymer.

The second set of simulations that were realized concerned the electromechanical activities of the “smart” guide wire. The simulation showed that the strain occurring mainly in the section near the base of the electrical field was the highest, and therefore corresponded to the electrostatic force. A comparison between the different compositions of the electroactive polymer under a constant electrical field of 30 V/μm is presented in Fig. 4 The advantage of using an all-organic composite is clearly demonstrated in the simulation, where the generated bending angle was higher than for the neat polymer. An electrical field six times higher will be needed to obtain the same strain for a pure polymer, which corresponds to the dielectric breakdown of the materials^21^. Figure 5 shows the generated displacement along the length of the guide wire, for different electrical fields and when the electrical excitation is applied to electrode 1 or 2. The simulation demonstrates the possibility to generate displacement in both directions, when the electrode has been sectorized.

## Materials and Characterization

The validity of the proposed design was investigated using a prototype guide wire developed to explore control and sensing approaches for use in medical procedures, especially for cardiac applications. The prototype of the “smart” guide wire was designed to achieve a workspace sufficient for this application.

In this study, a fluorinated terpolymer P(VDF-TrFE-CTFE) was used due to its high mechanical energy density. However, a large electrical field was needed (E > 150 V/μm) in order to reach sufficient strain levels (>2%)^19^, as is demonstrated by the simulation. The major drawback of electrostrictive polymers lies in the high electrical fields required. On the one hand, high electrical fields can be achieved at low voltages if the films are thin. On the other hand, the electromechanical transduction properties of any electrostrictive polymers are intrinsically regulated by the dielectric permittivity and Young modulus, Y, of the material^31^. These parameters directly control both the achievable active stresses and strains. In order to increase the dielectric permittivity of polymer materials, different methods have been developed. The main approach is based on the dispersion of filler in the polymer matrix. However, one of the typical drawbacks of the composite approach lies in a substantial decrease of the material’s dielectric strength (*E*_*break*_)^32^.

**Figure 6:**
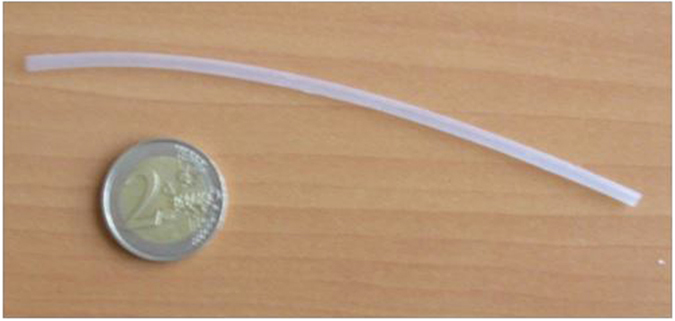
Extruded tube of P(VDF-TrFE-CTFE) with 15%wt DEHP.

The architecture of the “smart” guide wire can be described as a tube, and can thus be realized by extrusion. Commercially available terpolymer products do not comprise a plasticizer. In order to extrude an all-organic composite tube, a terpolymer with plasticizer was thus fabricated using a solution casting method. First, a glass plate was covered with a film of lecithin dissolved in cyclohexane (5 w/w% solution). A doctor blade (Elcometer) was then employed to create a film with a thickness typically between 80 μm and 180 μm. After drying at room temperature, the film was heated during 1 h at 70 °C to ensure complete evaporation of the solvent. Then, the film was cut in small pieces of 5 × 5 mm^2^. The tubings were extruded through a Laboratory Mixing Extruder (LME, Dynisco, Inc.) with a 0.476 cm outer die (o.d.) head and a 0.3175 cm mandrel tip at a mandrel temperature of 150 °C and a die temperature of 130 °C at 180 RPM. Next, the tube was cut into the desired length and recrystallized in an oven at the onset of the melting peak determined by a Setaram EVO 131 Dynamic Scanning Calorimetry (DSC). To realize a sufficient strain with the applicable voltages (lower than 10 kV), the thickness of the polymer had to be in the range of a few hundred microns. Figure 6 shows a picture of an extruded tube of P(VDF-TrFE-CTFE) with 15%wt DEHP. According to previous works, the highest electrostrictive behavior was found in 15% wt. modified DEHP samples^22^. Thus, this DEHP content was used in this study.

To evaluate the steerable properties of the fluorinated terpolymer films, a dedicated test setup, was built. On one side of the tube, a gold electrode was deposited by sputtering (Cressington 208 HR) through a mask. This made it possible to realize electrode 1 and 2. The inner electrode was created with the help of conductive grease. The sample was then connected to a commercially available guide wire in which an electric wire was manually integrated. The electric contact between the guide wire and the tube was assured with conductive glue.

**Figure 7:**
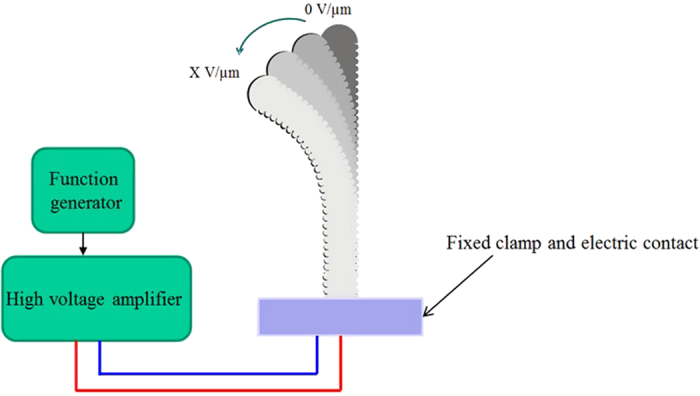
Schematic of the electromechanical characterization test setup of the “smart” guide wire.

The setup developed for characterizing the transverse vibration response of the polymer is shown schematically in Fig. 7. As seen in the Fig. 7, the “smart” guide wire to be tested was clamped in one end at a solid base (fixed) near the electric contact whereas the other end was free. When the polymer film was subjected to an electrical field, its expansion and contraction caused the free end of the tube to move and the movement was detected using an optical technique. In the current setup, a high-resolution camera was used to monitor the deflection (Canon 6D). The electrical field application was carried out with the Agilent 33220 A Function Generator and Treck power amplifier. All measurements were recorded in an open air environment (22 °C).

## Results and Discussion

In order to validate the design developed in Section 2, the electromechanical response was measured as a function of different stimuli.

### Morphing

**Figure 8:**
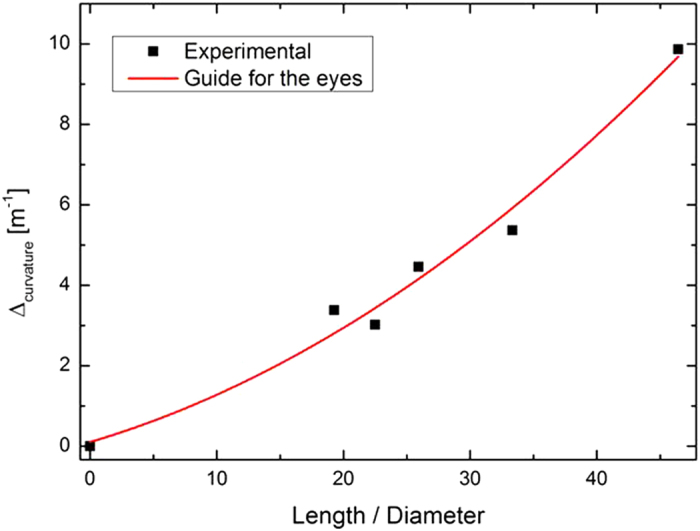
Experimental bending angles as a function of the electrical field.

To demonstrate the device performances of the all-organic composites, several tube actuators were fabricated with different ratios between their length and outside diameter. Figure 8 shows the measured curvature for an electrical field of 25 V/μm as a function of the length (L)/outer diameter (D_outer_) ratio of the tube. It is interesting to note that the achievable curvature for a constant electrical field was higher when the length/diameter ratio was maximal. This dependence was logical since the global stiffness of the structure was lower when the ratio was high^33^.

**Figure 9:**
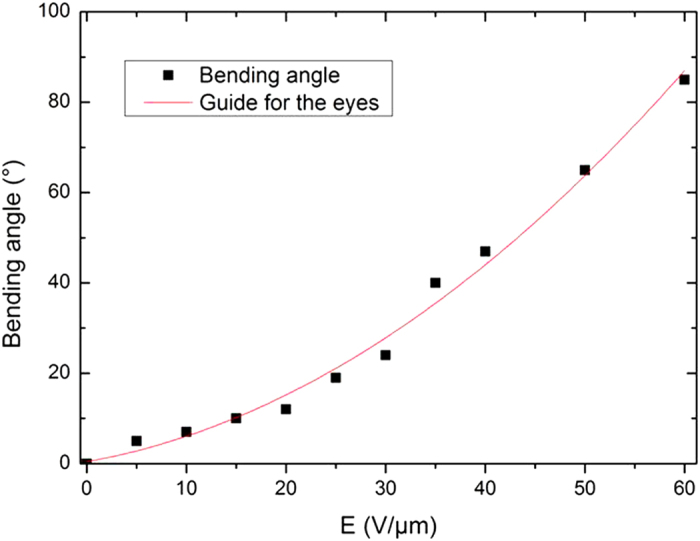
Smart guide wire for two electrical fields.

**Figure 10:**
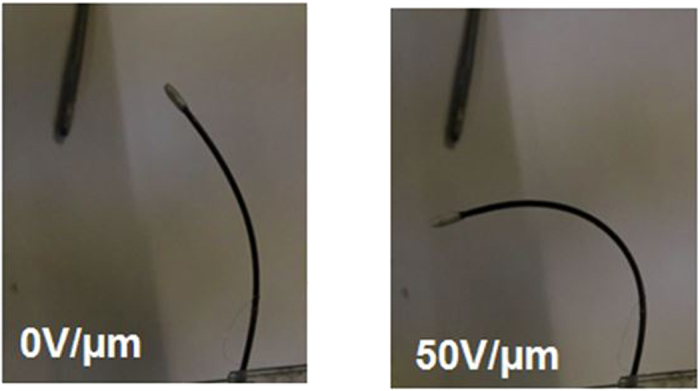
Bending angle as a function of time under a step electrical field of 20 V/μm.

Figure 9 shows the bending angles generated as a function of a static electrical field at room temperature for a sample with a length of 65 mm, an inner diameter of 1 mm and an outer diameter of 1.4 mm. A quadratic dependence between the bending angles and the electrical field can be noted. This observation corresponds to the theory that shows such a dependence between the strain and the electrical field in electrostrictive materials^34^,^35^. Moreover the bending angle produced by the proposed design corresponds to the typical angle available in the human body^28^, making it possible to easily navigate the “smart” guide wire through the blood vessels. This function of steerability is relatively important to the development of the MIS procedure^26^. To illustrate the generated displacement, pictures of the “smart” guide wire for two electrical fields are given in Fig. 10. It should be noted that the maximum electric filed used in experiment equal 50 V/μm resulting in a voltage of 10 kV cannot be considered to be safe for human body. A simple solution is to reduce the thickness of the electrostrictive material in order to significantly decrease the applied voltage. This can be achieved based on a multilayer structure consisting several thin electrostrictive polymers sandwiched between the electrodes. The multilayer architecture allows to considerably decrease the thickness of the whole actuator compared to the tube architecture. A fabrication of such multi-electrostrictive layers can be carried out using ink-jet printing technology^36^. This aspect should be investigated in future work.

**Figure 11:**
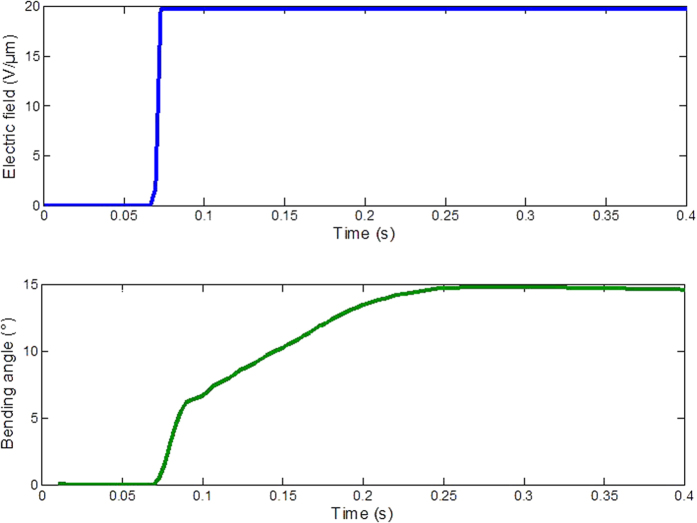
Temporal evolution of the mechanical stress and generated charge.

Figure 11 shows the step response of the prototype that was actuated by an electrical field of 20 V/μm. There were clearly two parts to the response. The fast part accounted for about 52% of the total measured bending angle. The slow part took about 100 ms to reach 90% of the total measured bending angle and was attributed to creep^30^,^37^,^38^. The time response of the material was higher than for other electrostrictive polymer such as IPMC or conductive polymers with typical time responses above 2s^39^. Moreover, the time response was compatible with the application since the reaction time for humans is about 180 ms as presented in section 2 (Requirements and working principles).

### Force feedback

A steerable property is not the only function requested for the development of a “smart” guide wire. For example, minimally invasive surgery operations are more and more often performed using a robot through small incisions^40^. Force feedback is widely assumed to enhance performances in robotic surgery. However, current commercial robotic surgery systems provide no force feedback from the instruments, yet surgeons have demonstrated the ability to use these systems to perform delicate procedures such as coronary artery bypass grafting^41^,^42^. Despite this demonstrated ability to work without force information, the dexterity with current minimally invasive instruments, whether manual or robotic, is clearly less than optimal. Furthermore, the implementation cost of force sensors that allow force feedback is prohibitive, due to the stringent design requirements imposed by the surgical environment^43^.

Due to its unique properties, the P(VDF-TrFE-CTFE) composite employed to realize the “smart” guide wire can be used as a force sensor. In fact, by assuming a linear relationship between the electrical field I and the polarization (P = (ɛ− ɛ_0_)E, where ɛ is the dielectric constant), it can be shown that the linearized constitutive equations for a pure electrostrictive material, i.e., without piezoelectric behavior, can be expressed using the Voigt notation^31^,^44^ according to


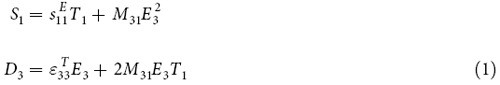


where, S, T and D denote the total strain in the material, the stress component, and the electric displacement, respectively, 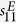 is defined as the elastic compliance under constant stress and *M*_*31*_ is the electrical field-related electrostrictive coefficient. It should be noted that the above assumption is fully valid for the investigated fluorinated terpolymer since a linear variation in the polarization versus electrical field magnitude up to 50 V/μm has been demonstrated^45^.

As shown in Eq. 1, electrostriction is generally defined as a quadratic coupling between the strain and the polarization. When working in sensor mode, the electrostrictive polymer is subjected to a DC-biased electrical field. Since the polymer is not piezoelectric, it is necessary to induce polarization with this DC bias in order to obtain a pseudo-piezoelectric behavior^35^,^46^. The current induced by the mechanical stress can be expressed as:


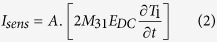


The product “2.M_31_.E_DC_” corresponds to the pseudo-piezoelectric coefficient. The generated current is proportional to the mechanical excitation, and it is thus possible to measure the force applied by the surgeon to the patient so as to obtain force feedback information.

**Figure 12:**
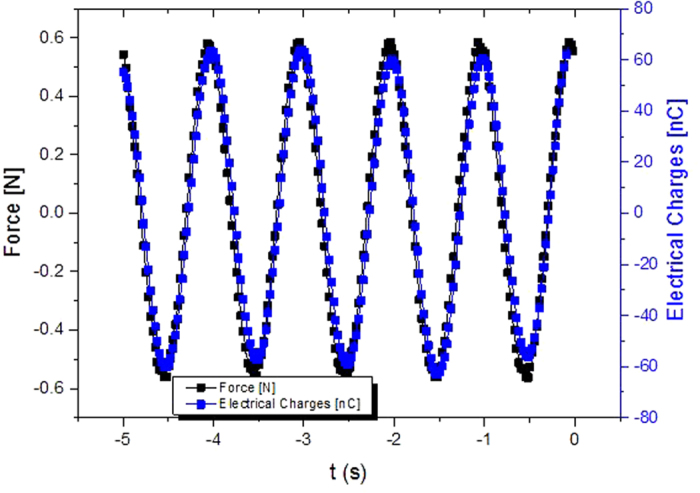
Electrical response in pseudo-piezoelectric mode as a function of the mechanical force.

Figure 12 displays the temporal evolution of the stress and the electrical response of an electrostrictive polymer, working in pseudo-piezoelectric mode (E_DC_ = 6 V/μm). The generated electrical charge and the force were in phase; in fact, the transported charge was the key governing the electromechanical conversion. It can be clearly seen in equation (Eq. 1) since the electrical displacement was proportional to the time derivative of stress.

**Figure 13:**
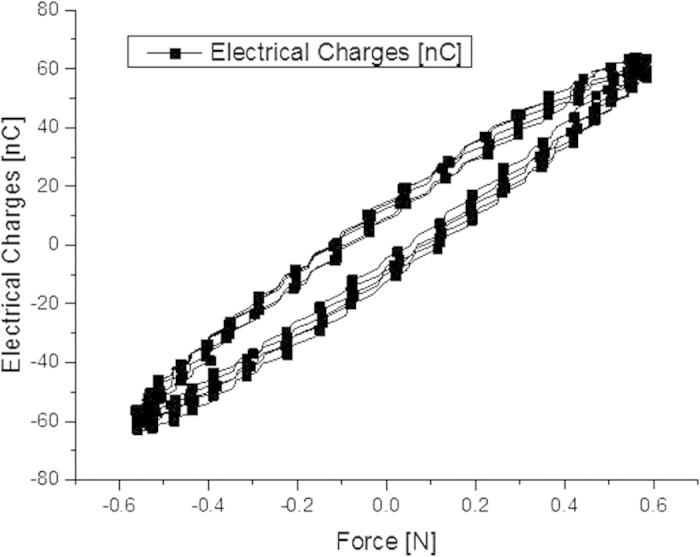
Electrical response in pseudo-piezoelectric mode as a function of the mechanical force.

Figure 13 plots the electrical charge as a function of the force. The amount of transported charge was calculated by the time-integration of the measured current. A linear dependence between the electrical charge response and the mechanical force solicitation is clearly visible. This linear dependence exists theoretically as demonstrated in Eq. 2.

**Figure 14:**
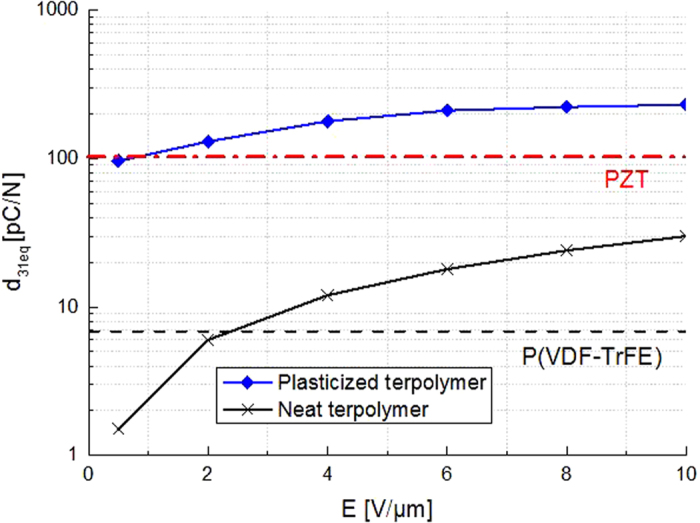
Pseudo-piezoelectric coefficient at 1Hz as a function of the static electrical field.

The equivalent piezoelectric coefficient was provided to facilitate the comparison of the electromechanical activities of an electrostrictive material with a common piezoelectric material. It is related to the electrostrictive coefficient according to *d*_*31*_ = *2.M*_*31*_.*E*_*DC*_. Figure 14 gives the evolution of the piezoelectric coefficient as a function of the static electrical field (E_DC_). One can see that all organic composites exhibited substantial piezoelectric coefficients equivalent to those of the piezoelectric ceramics for an electrical field lower than 10 V/μm. At this value of the electrical field, the neat polymer only had a piezoelectric coefficient equivalent to that of P(VDF-TrFE). These results demonstrate the pros of operating in pseudo-piezoelectric mode as this facilitates the design (same material for sensing and actuating) and the sensor sensitivity is equivalent to that of a piezoelectric ceramic. For example, a typical amplitude of force applied in the case of MIS is comprised between 1N and 10N^47^, so the quantities of generated electrical charges are enough to be detected by the material for an electrical field of 1 V/μm.

## Conclusion

This article presents the modeling and development of an electrostrictive tube for the steering and force feedback of a guide wire. An electrostrictive tube was desired to equip with the distal tip of a guide wire for endovascular navigation in order to reach the target regions inside blood vessels and through the tortuously curved pathways of blood vasculature. The proposed material is considered to be an excellent candidate for endovascular navigation thanks to its high flexibility and low current consumption. Furthermore, the developed electrostrictive polymer can be configured in both sensor and actuator modes, giving it a possibility of miniaturization to very small endovascular sizes. Such properties are extremely interesting for performing a multifunctional medical tool, especially in minimally invasive surgery where force or curvature sensor can be easily incorporated.

Future works focus on fabrication process of multi-electrostrive layers using ink-jet printing. This method allows to drastically decrease the material thickness, leading to enhanced electromechanical response under lower applied electric field. Another aspect of this work is to take into account the patient’s safety by choosing biocompatible plasticizes or adding a protection system into the catheter.

## Additional Information

**How to cite this article**: Ganet, F. *et al.* Development of a smart guide wire using an electrostrictive polymer: option for steerable orientation and force feedback. *Sci. Rep.* **5**, 18593; doi: 10.1038/srep18593 (2015).

## Figures and Tables

**Table 1:** Ansys parameters and variables.

Name	Value	Description
D_outer_	1.4 mm	Outer diameters
D_inner_	1 mm	Inner diameters
L	60 mm	Extrusion Length
E	30 V/μm	Electric field

**Table 2:** Electromechanical properties of electroactive polymers at room temperature.

Type	Y (MPa)	ɛ_r_ at 0.1Hz and E = 20 V/μm	M_31_ (m^2^/V^2^)
P(VDF-TrFE-CTFE)	100	30	3.10^−18^
P(VDF-TrFE-CTFE) + 15wt%DEHP	30	400	38.10^−18^
